# Microwave-assisted of new derivatives of polyimine conjugated polymer based on Schiff base: synthesis, characterization, and photo-physical properties as a photoluminescent materials

**DOI:** 10.1038/s41598-023-46051-w

**Published:** 2023-10-31

**Authors:** El-Refaie Kenawy, Ahmed R. Ghazy, Hala F. Rizk, S. Shendy

**Affiliations:** 1 Polymer Research Group, Chemistry Department, Faculty of Science, Tanta University, Tanta, 31527 Egypt; 2 Laser Laboratory, Physics Department, Faculty of Science, Tanta University, Tanta, 31527 Egypt

**Keywords:** Materials science, Optics and photonics

## Abstract

The condensation of pyrrole-2,5-dicarbaldehyde **(1)** with 5-(2-amino-4-phenylthiazol-5-yl)-4-phenylthiazol-2-amine **(2)** and/or 5-(4-Amino-phenyl)-4-phenylthiazol-2-amine **(3**) gave new poly(*Z*)-*N*-((5-(iminomethyl)-*1H*-pyrrol-2-yl)methylene)-5-(2-((*E*)-(5-(iminomethyl)-I-pyrrol-2-yl)methyleneamino)-4-phenylthiazol-5-yl)-4-phenylthiaol-2-amine **(P1)** and/or poly(*E*)-*N*-((5-(iminomethyl)-*1H*-pyrrol-2-yl)methylene)-5-(4-((*E*)-(5-(iminomethyl)-*1H*-pyrrol-2-yl)methyleneamino)phenyl)-4-phenylthiaol-2-amine **(P2)** as a novel conjugated polymer by microwave irradiation and traditional heating.. It is evident that the microwave irradiation technique quickly raised the molecular weight of polyimines. In addition to quantifying the molecular weight of the resultant polyimines. All the polyimines were characterized using FTIR, XRD, H1NMR, TGA, and DSC. The optical characteristics of polyimine derivatives were investigated using a UV–Vis spectrophotometer. The absorption spectra showed a main absorption band around 372 nm for polyimine **(P1)** and 381 nm for polyimine **(P2)**. The optical energy was calculated and found to be 2.49 and 2.68 eV. The photoluminescence of the polyimine derivatives was measured and analyzed by spectrofluorometer and Laser photoluminescence experiment and the emission color was studied using CIE graphs. The fluorescence spectra showed an emission peak at 548 nm for polyimine **(P1)** with yellow green color in CIE graph, while for polyimine **(P2)** the emission band was located at 440.5 nm with blue color in CIE graph. Photoluminescence quantum yield PLQY was measured for the polyimine P1 and P2 in both liquid and Solid states and indicated the AIE behavior of the polyimines. TD-DFT simulations were applied to the polyimine derivatives where the structures were geometrically optimized and the spectroscopic characterizations were evaluated.

## Introduction

Azo polymers have distinct UV/Vis absorption spectra and a wide range of colors due to the large number of vibronic states in each energy level. These compounds have therefore been employed as pigment, as well as more recently in electrical applications as photochromic, photo-switching, and optical data storage^1–3^. Researchers have recently put forth creative alternatives, such as azo-based fluorophores for organic and polymeric light emitting devices, for sensing metals, and as bio-analytes, in order to address the current issue of developing novel organic simple and inexpensive luminophores^4–6^.

Traditional fluorescent materials were frequently used as dopants to produce high quantum yield emissions. These materials were doped sparingly to avoid the well-known phenomenon of aggregation-caused emission quenching (ACQ), which is linked to the formation of less emissive species like exciplexes and excimers in order to boost emission intensity.

Recently, it was discovered that a still-small number of organic chromophores emit more effectively in the aggregated state than in solution. In 2001, Tang and colleagues created the first derivative, which is nearly non-emissive in solution but is significantly emissive when aggregated^7^. The aggregation-induced emission (AIE) effect was used to describe this phenomenon^8^. The most frequently proposed explanation for this phenomenon is the restriction of intramolecular rotation (RIR)^9^. In solution, unconstrained intramolecular rotation creates a non-radiative relaxation pathway for the excited state to decay. The spin can be slowed down or stopped to bring back molecular fluorescence. AIE fluorogens have recently attracted a lot of research attention due to their distinctive optical characteristics and wide range of applications, as they are crucial for the creation of effective solid-state devices^10,11^.

The direct transformation of fundamental chemical building blocks into usable products is the main objective of polymer synthesis. Numerous investigations on -conjugated polymers have been conducted. Poly (azomethine)s, known as polyimines or Schiff base polymers. These polymers are mainly obtained by polycondensation reactions between diamines and dialdehydes monomers. Polyimines and they classified as “high-performance” conjugated polymers due to their great mechanical strength, thermal stability, chemical inertness, good electrical conductivity, fluorescence, and photo-acoustic properties^12^. These amazing properties are greatly influenced by the polymer chain's alternating single (sp hybridization) and double (sp2 hybridization) bonding^13^. Additionally, they have low dielectric constants, little thermal expansion, little weight, outstanding flexibility, straightforward film production, and excellent radiation resistance^14^. They are therefore frequently employed in the electric^15^, photonics^16,17^, electro-optical industries^18^. membrane for separating gases and chemicals^19–21^, Proton exchange membranes^22,23^ and the aerospace industry^24^. The synthesis of two-dimensional (2D) and three-dimensional (3D) covalent organic frameworks (COFs) has extensively used the imine bond produced by the condensation of an amine and an aldehyde^25^. Imine COFs have received a great deal of attention in the last ten years due to their high chemical and thermal stability, porosity and crystallinity^26^, as well as their potential applications in gas storage, ion separation, semiconductors, proton conduction, luminescence, catalysis, and energy conversion^27^.

Thiazoles and pyrroles are two of the most useful substances for use in agriculture and medicine^28^. Due to its antiallergic, antibiotic, anticancer, anti-HIV, anti-inflammatory, antifungal, antibacterial, and antioxidant effects, thiazole is chosen over other chemicals^29^. Poly(heterocycles) (polypyrroles1, polythiophenes2,3, and others4-6) and their copolymers have changed the way scientists think about developing organic electronics such as semiconductors, photovoltaic devices, and sensors^30–33^.

The employing of neoteric TD-DFT techniques (DMol3 and CASTEP approaches) to investigate the structure of polymer matrix, copolymer phase stability, and nanocomposite compounds is discussed^34,35^. Little emphasis has been paid to the application of this comprehensive energy-based technique for the estimate and study of spectroscopic characteristics. The potential energy of the HUMO and LUMO states are studied geometrically in this article utilizing a restricted programming language^36,37^. In order to attain high degrees of precision, it is intended to show that the same atomistic modeling tools may be consistently used throughout the experimental investigation^38^. The electron–ion potential is expressed by ab initio pseudopotentials in either standard-memorizing or ultrasoft formulations. The appropriate charge intensity, Kohn–Sham wave functions, and a conscience-consistent procedure are derived in accordance with the direct energy reduction. In particular, density mixing and conjugate approaches are used. A powerful DFT electron could be used to depict the shape of systems with a limited population^39,40^. The crucial variables that affect the convergence of the measurements are the copolymer and composite compounds with various k-points utilized for precise Brillouin zone integration and the plane waves cut-off that provides the base set size^41^.

In recent years, many researchers have worked on high performance AIE polymers. Xu et al. reported the synthesis of AIE polymers with unchanged emission wavelength by ring-opening (co)polymerizations of 4-(triphenylethenyl)phenoxymethyloxirane (TPEO) and other epoxides or phthalic anhydride with a quantum yield of 39.1%^42^. Caruso et al. successfully synthesized an AIE based on a phenylenevinylene (PV) and a dicyano-PV with a PLQY of 75% in the solid phase^43^.

In this work, we aimed to synthesize a novel aggregation induced emission (AIE) Schiff base polymers based on polycondensation reactions between diamines and dialdehydes (polyimine). These polymers were synthesized by conventional heating and microwave methods. The structural characterization of the polymers was studied using FT-IR, ^1^H NMR, UV–Vis, and Thermal analysis. photophysical properties were studied for these polymers and the photoluminescence performance was also studied in terms of fluorescence, laser photoluminescence and quantum yield for the polymers in solution and solid states. TD-DFT simulations were also performed on synthetic polymers.

## Experimental

### Chemistry

The used chemicals were obtained from Sigma-Aldrich and used without further purification, and the solvents were of spectroscopic grade.

### Instrumentation

Temperatures were not corrected for melting points, which were measured using a Gallen Kamp melting point instrument. On the Jasco FT/IR-4000 spectrometer, KBr pellet infrared spectra were captured. On a Biochrom Libra S50PC controlled scanning UV/Vis spectrophotometer, encompassing the wavelength range of 190-1100 nm, UV–Vis absorption spectra were acquired using 10 mm quartz cells. On a Bruker AC spectrometer, 1H NMR spectra were captured at 400 MHz in CDCl3 at 25 °C. Chemical shifts are presented in ppm as values and were measured against TMS as an internal standard. Thermogravimetric Analyzer (TGA) was performed using a Perkin Elmer TGA 4000 (United States) at Faculty of science, Tanta University. Cu K (= 1.540 A) was used to produce an XRD pattern using a Rigaku X-ray diffractometer. The 2's scanned range was between 5 and 80. Using a JEOL JSM-IT100 operating at 20 kV over samples, SEM pictures were produced. Using a BioRad ES100 SEN coating equipment, samples were given a light gold coating. Site = ECA500; Spectrometer: Datum BL DELTA2_NMR. Using a microwave, Aurora Transform 800 digestion 10 Vessels (9 Standard and 1 Sensor), 800 psi Operating Pressure, 250 °C Operating Temperature, 50 mL Vessel Volume, 1200 W Microwave, 2450 MHz Magnetron. Thin layer chromatography (TLC), using n-hexane/ethyl acetate (1/1 by volume) as the eluent, was used to track the development of the reaction. Pyrrole-2,5-dicarbaldehyde **1**, 5-(2-Amino-4-phenylthiazol-5-yl)-4-phenylthiazol-2-amine 2 and 5-(4-Aminophenyl)-4-phenylthiazol-2-amine** 3** were synthesized by using our published data^44^.

### General procedure for synthesis of polyimines P1 and P2

#### Method (A): conventional heating

Polycondensation of pyrrole-2,5-dicarbaldehyde **1** (0. 3 g, 2.5 mmol) and 5-(2-amino-4-phenylthiazol-5-yl)-4-phenylthiazol-2-amine **2** and/or 5-(4-Aminophenyl)-4-phenylthiazol-2-amine **3** (2.5 mmol) were carried out using 20 mL ethanol as solvent under nitrogen atmosphere. The reaction mixture was heated for 10 h under reflux. Polymer was precipitated after 30 min from refluxing reaction. The formed precipitate was separated from the solution by filtration, washed three times with cold ethanol, and dried in vacuum.

#### Method (B): microwave‐assisted irradiation technique

Similar to technique A's procedure, this one involved copping the mixture in closed vessels and irradiating it in a microwave oven with 800 Microwave digesting systems. The digestion procedure was optimized at 23 min at target temperature 40–70 °C. Polymer was precipitated and the formed precipitate polyimine **P1** and/or **P2** were separated from the solution by filtration, washed three times with cold ethanol, and dried in vacuum.

Polyimine **P1:** brown powder; m.p. 92–95 °C, yield: 0.5 g (50%). IR (KBr) *v*/cm^−1^: 765.6 (C–S), 3431.7 (CH), 3055.66 (N–H), 1622.8 (C=N). ^1^H NMR (CDCl_3_, 400 MHz) δ (ppm): 2.5 (s, exch., 4H, NH2), 7.37–7.5 (m, 10H, Ar–H), UV–Vis: (CHCl_3_) λmax, nm 303, 376.

Polyimine **P2:** orange powder; m.p 185–190 °C, yield: 0.56 g (63.4%). IR (KBr) *v*/cm^−1^: 862 (C–S), 3423 (CH), 3268.7 (N–H), 1617.9 (C=N). ^1^H NMR (CDCl_3_, 400 MHz) δ (ppm): 6.9–7.4 (m, 10H, Ar–H), 8.82 (s, CH=N). UV–Vis: (CHCl_3_) λmax, nm 335, 384.

### A computational investigation of synthetic polymers in the form of single molecules existing in a gaseous state

Estimates of the molecular structure and frequency dimensions of polyimines P1 and P2 in the gas phase were made using the TD-DFT/CASTEP and TD-DFT/DMOl3 simulation methods. TD-DFT/CASTEP and TD-DFT/DMOl3 software were used to estimate the GGA functional correlations, Perdew–Burke–Ernzerh (PBE) exchange, pseudo-conserving norm, and DNP base set for the free molecules^45,46^. The calculations of the structural matrix simulation led to the conclusion that the cut-off energy for plane waves is 550 eV. TD-DFT/DMol3 and TD-DFT/CASTEP frequency computation computations at the gamma point were used to examine the optical and structural or spectroscopic properties of polyimines P1 and P2. It was demonstrated that the functional non-local interchange of Becke's law may be used to generate the functional B3LYP of polyimines P1 and P2 in the gas phase^47^. Measurements of the infrared vibrational frequency were carried out using WBX97D/6-311G. Using the Gaussian 09W software system, geometric attributes, energy, vibration modes, and the ideal configuration picture were examined^48^. The B3LYP approach for TD-DFT computations is based on WBX97XD/6-311G, as demonstrated in earlier studies, and has produced outstanding results for experimental discoveries and structural spectrum correlation^49^. In order to assign Gaussian and TD-DFT/CASTEP computations for polyimines P1 and P2 models in the gas phase, the Gaussian Potential Approximation System (GAP) explains the concurrent usage of many independent uncertainty models as well as the overall power and derivatives model.

### Methodology

Polyimines P1 and P2 undergo optical and photoluminescence investigations as liquid and solid materials. Firstly, P1 and P1 were dissolved in ethanol, chloroform, and DMF with a concentration of 1 × 10^−4^ mol/L. Then a dip casting method was used to deposit the polymers on glass substrate forming the polymeric films.

The liquid samples were measured for their absorption and fluorescence spectra in a 1 × 1 cm quartz cell. And the quantum yield was measured for the samples in the different solvents relative to fluorescein dye (as the standard). Laser photoluminescence investigation was applied to the solid samples using a He-Cd laser with 325 nm wavelength and 150 mW power as an excitation source and the spectrum was recorded using HoRiBA (IHR 320) spectrum analyzer with a computerized CCD camera.

## Results and discussion

**Scheme 1:**
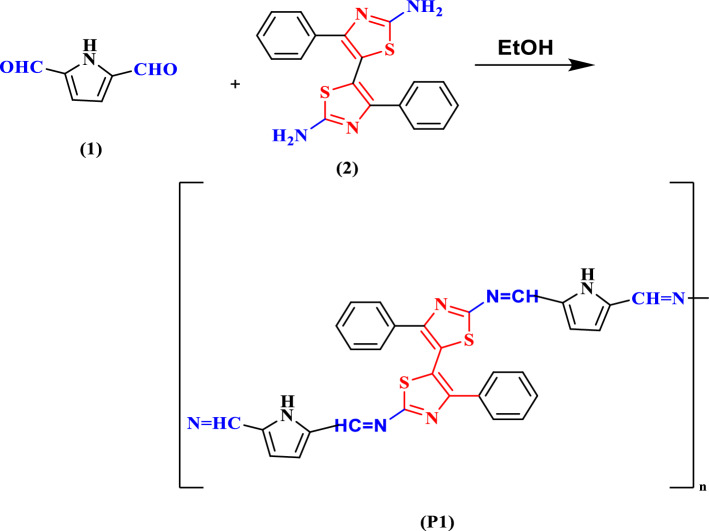
Synthesis of polyimine P1.

**Scheme 2:**
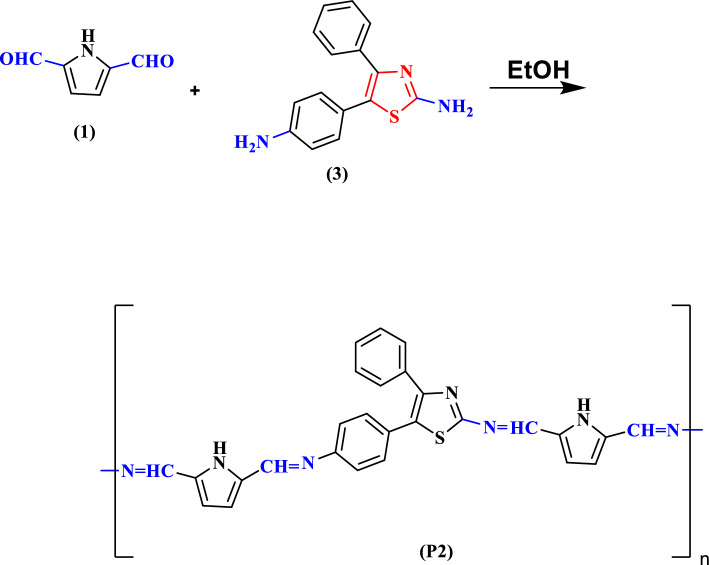
Synthesis of polyimine P2.

A new series of polyimines were synthesized by conventional heating and microwave irradiation technique by condensation of pyrrole-2,5-dicarbaldehyde **1** with 5-(2-amino-4-phenylthiazol-5-yl)-4-phenylthiazol-2-amine **2** and/or 5-(4-Amino-phenyl)-4-phenylthiazol-2-amine **3** in ethanol to yield poly(*Z*)-*N*-((5-(iminomethyl)-*1H*-pyrrol-2-yl)methylene)-5-(2-((*E*)-(5-(iminomethyl)-I-pyrrol-2-yl)methyleneamino)-4-phenylthiazol-5-yl)-4-phenylthiaol-2-amine **P1** and poly(*E*)-*N*-((5-(iminomethyl)-*1H*-pyrrol-2-yl)methylene)-5-(4-((*E*)-(5-(iminomethyl)-*1H*-pyrrol-2-yl)methyleneamino)phenyl)-4-phenylthiaol-2-amine **P2** (Schemes 1, 2). Due to its insolubility in ethanol, the beginning of the polymerization process by conventional heating occurs after 30 min. This early precipitation results in low molecular weight as black and orange precipitates, respectively. The molecular weight of the prepared polyimines was measured using static light scattering technique and was summarized in Table 1.

**Table 1:** At a wavelength of 650 nm and at room temperature, polyimine dissolved in chloroform was measured for its second virial coefficient, molecular weight, radius of gyration, and refractive index increment.

		dn/dc (mL/g)	M_w_ (g/mol)	R_G_ (nm)	A_2_ (mol cm^3^/g^2^)
P1	C. Heating	13.06	3496	828	7.185
	Microwave	9.54	5484	734	3.99
P2	C. Heating	10.03	9450	2846	80.13
	Microwave	16.08	10,622	4350	50.931

All the newly synthesized conjugated polymers are characterized FTIR, UV, ^1^H NMR, TGA, XRD and EDX.

In comparison to traditional heating and microwave technique the reactions to be completed in just a few minutes (23 min) instead of (10 h). Furthermore, the synthesised compounds were directly generated in pure form and had higher molecular weight.

### Static light scattering

The refractive indices of the polymers under study have been determined at concentrations that would be employed in light scattering studies in order to calculate the molecular weight Mw, second virial coefficient A2, and radius of gyration RG accurately^50,51^. In this study, chloroform was used to dissolve the polyimine p1 and p2, and refractive index measurements were made at concentrations of [(1, 2, 3, 4) × 10^−4^ and (1, 2, 3, 4) × 10^−5^ g/mL] and the following equation has been used to compute the refractive index increment^52,53^.

$$\left. {\frac{dn}{{dc}}} \right|_{c \to 0} = \mathop {\lim }\limits_{c \to o} \left( {\frac{{n - n_{o} }}{c}} \right)$$

where n, no, and c stand for the solution refractive index, the solvent refractive index, and the concentration, respectively. Table 1 lists the derived values for the refractive index increment dn/dc.

**Figure 1:**
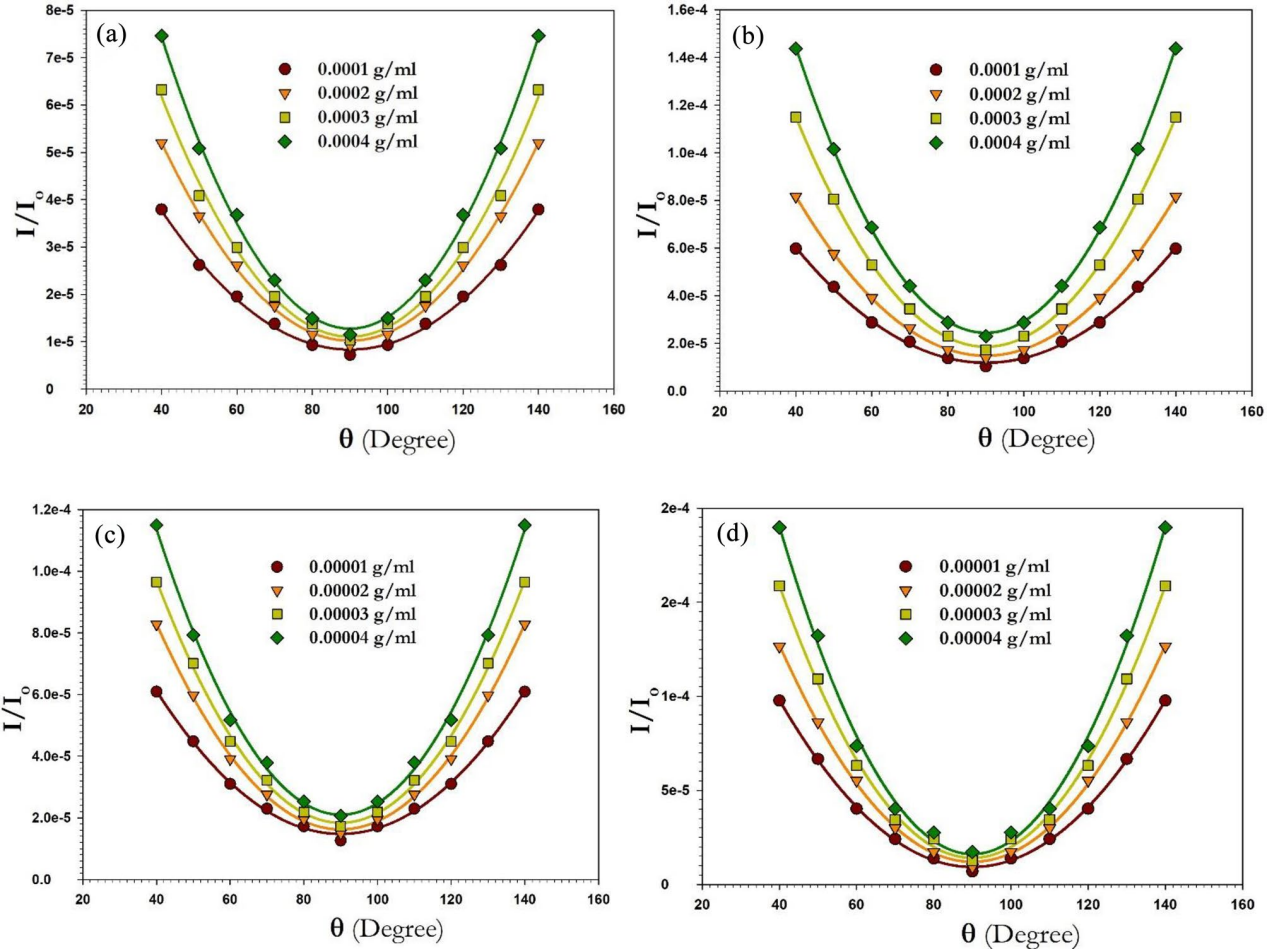
The angular distribution of the scattering intensities of (**a**) P1 conv. H, (**b**) P1 microwave, (**c**) P2 conv. H., and (**d**) P2 microwave.

Using a photomultiplier tube (Oriel Instruments, model 77,344, powered by an Oriel power supply, model 70,705) and a Nd-YAG laser with a wavelength of 650 nm and power of 5 mW as the light source, the angular distribution of the scattered light intensity shown in Fig. 1 was measured at angles ranging from (40°–140°) for the various concentrations of polyimine p1 and p2 dissolved in chloroform.

**Figure 2:**
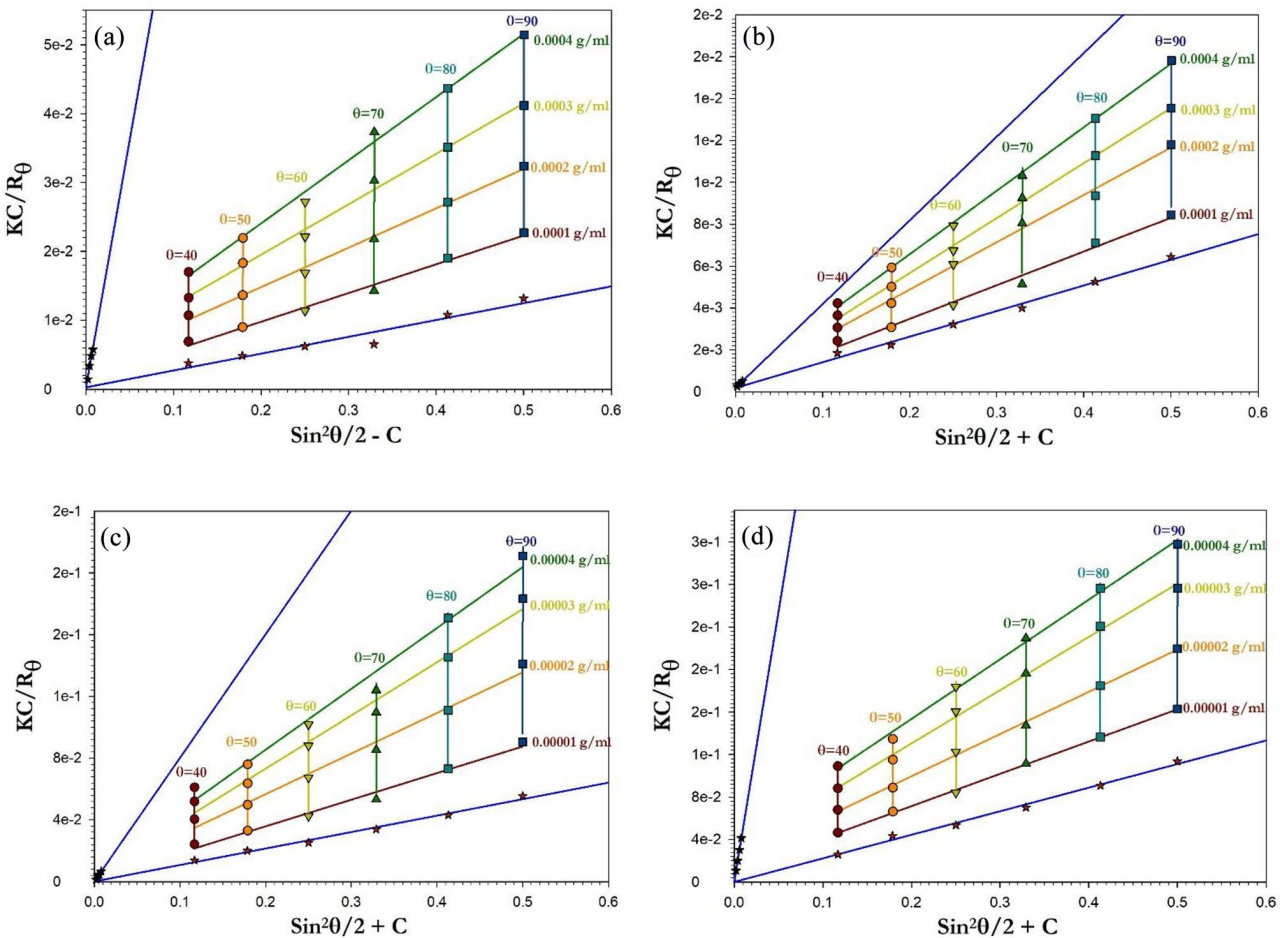
The construction of Zimm plot for (**a**) P1 conv. H, (**b**) P1 microwave, (**c**) P2 conv. H, and (**d**) P2 microwave.

The Zimm plot^54^ (Fig. 2) was created using the fundamental equation of static laser light scattering, and the values of the scattering parameters are provided in Table 1 together with the molecular weight (Mw), second virial coefficient (A2), and radius of gyration (RG)^55–58^.

$$\frac{Kc}{{R_{\theta } }} = \frac{1}{{M_{w} }}\left[ {1 + \left( {\frac{{16\pi^{2} }}{{3\lambda^{2} }}} \right)R_{G}^{2} \sin^{2} \left( {\frac{\theta }{2}} \right)} \right] + 2A_{2} c$$

where

$$K = \frac{{2\pi^{2} n_{o}^{2} }}{{\lambda^{4} N_{A} }}\left( {\frac{dn}{{dc}}} \right)^{2} \left( {1 + \cos^{2} \theta } \right)$$

$$R_{\theta } = \frac{{I_{\theta } r^{2} }}{{I_{o} V}}$$

where r is the distance between the scattering point and detector, V is the scattering volume, is the scattered light wavelength, $N_{A}$ is Avogadro's number, is the scattering angle, $n_{o}$ is the solvent's refractive index, $I_{\theta }$ and $I_{o}$ are the intensities of the scattered and incident light, respectively^59^.

### Spectra characterization of polyimines

**Figure 3:**
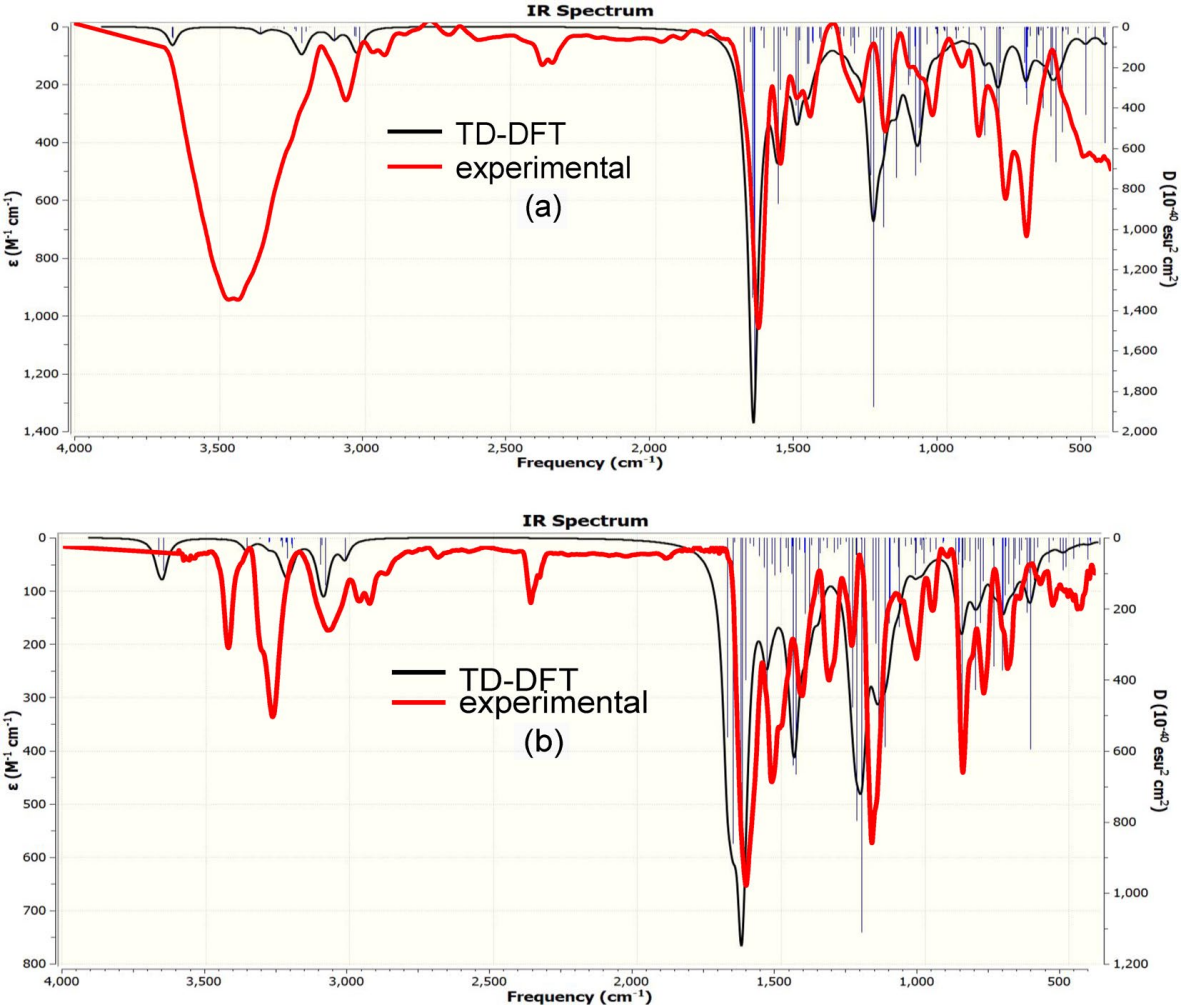
FT-IR spectrum for polyimines (**a**) P1 and (**b**) P2.

The FTIR of polyimines P1 and P2 (Fig. 3) showed bands at (3431.7, 3423) cm^−1^ characteristic of (C–H) stretching of benzene ring and bands at (1622, 1617.9) cm^−1^ characteristic of (CH=N) of azomethine linkage, respectively with absent of band at 1700 cm^−1^ corresponding to CHO group.

**Figure 4:**
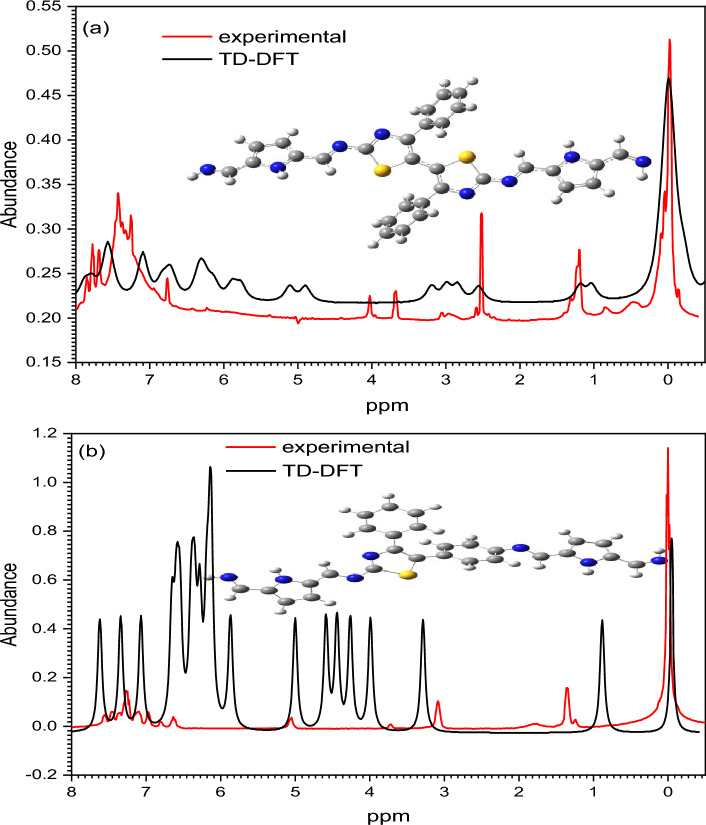
^1^H NMR spectrum of the polyimines (**a**) P1 and (**b**) P2.

The ^1^H-NMR spectrum of the polyimine P1 and P2 (Fig. 4) showed peaks at 8.36 (–CH=N–), three wide peaks positioned at 7.6, 7.5, 7.4, 7.3 and 7.0 ppm (aromatic protons from carbazole and phenyl rings).

### Thermogravimetric analysis (TGA) of polyimine

**Figure 5:**
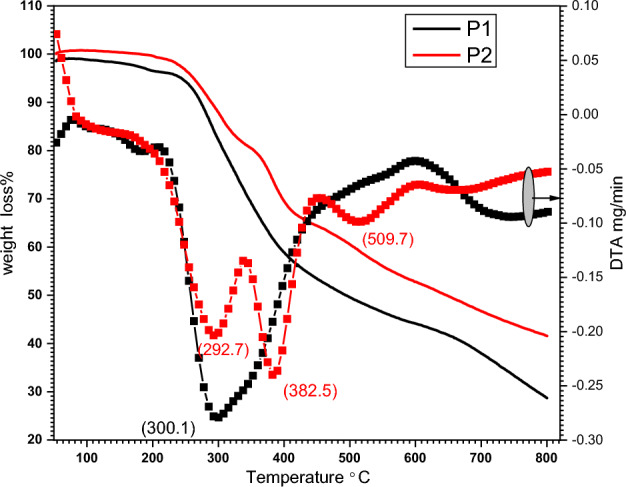
The thermal behavior (TGA and DTA) for polyimines **P1** and **P2.**

The thermal stability of the polyimines **P1** and **P2** were characterized by TGA. Samples 5–10 mg was located into alumina crucibles and scanned (from 50 to 800 °C) with rate 30 °C/min surrounded by nitrogen atmosphere with a flow rate of 20 mL/min, and their corresponding weight loss of 5% and 2% respectively up to 250 ^o^C were all determined from original TGA curves (Fig. 5). The mass loss for polyimines **P1** and **P2** in this region corresponds to the lattice water molecules loss and the decomposition of coordinated water molecules^49^. The TGA pattern of polymer **P1** showed a single step of degradation, while **P2** showed three steps of degradation. P1 started to decompose at 245 °C while P2 showed T_onset_ at 235 °C. Losing the 50%, weight loss of **P1** were observed at T_50%_ 493.9 °C and 647.9 °C for **P2** which indicate that **P2** is thermally stable than **P1**.

From DTG curves, **P1** showed maximum weight of decomposition at 800 °C while **P2** showed T_peak_ at 292.7, 382.5 and 509.7 °C. The total weight loss at 300 °C for polymer **P1** was 70% while P**2** displayed 57% of weight loss.

### EDX studies

**Figure 6:**
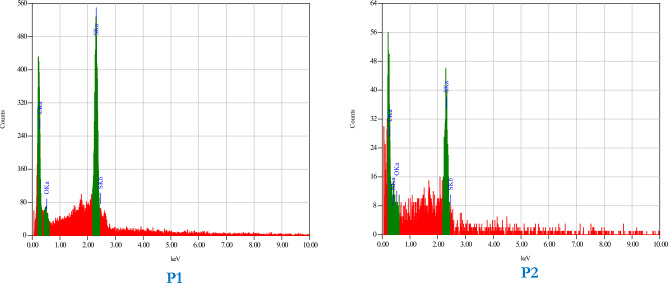
EDX spectroscopy of polyimine p1 and p2.

Elemental analysis was performed by applying EDX spectroscopy on polyimines **P1** and **P2** (Fig. 6). There were only the main elements which are carbon, nitrogen, oxygen and Sulphur without any impurities.

### TD-DFT molecular simulation

**Figure 7:**
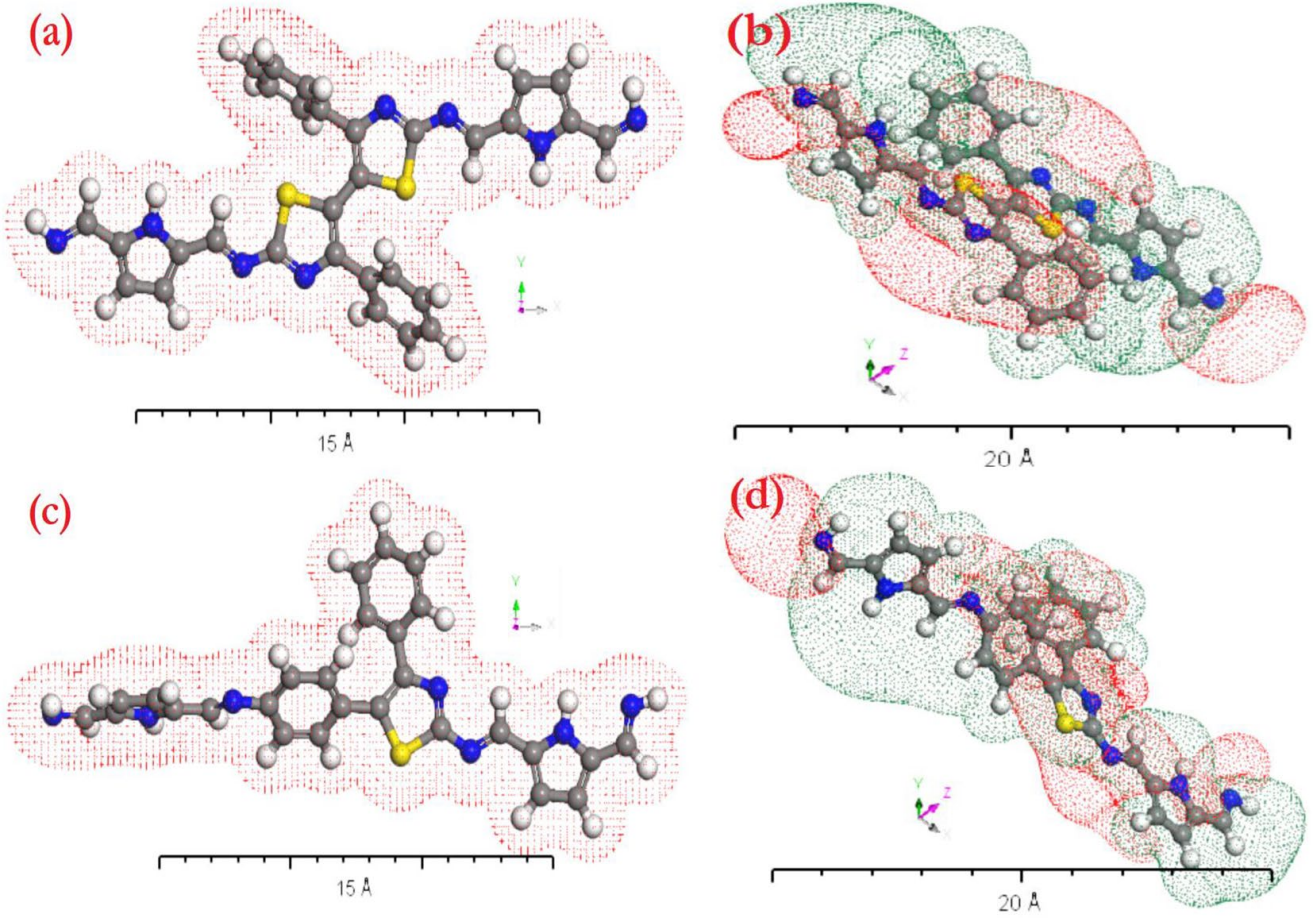
(**a**,**c**) Electron density of polyimine p1 and p2 gas phase, (**b**,**d**) Potential of polyimine p1 and p2 gas phase by the applications of TD-DFT/DMOl^3^ programs.

Electron density and electrostatic potential were used to analyze the polyimine p1 and p2 molecules' gaseous phase similarity features. The geometry for polyimine p1 and p2 was optimized using the PWC function in the DFT-DMOl3 computations. The study of gaseous phase electron systems employed electron density. On the other hand, by displaying the potential diagrams, it was possible to analyze the potential expansion of the polyimine p1 and p2 gas phases. The electron density and electrostatic potential studies provided evidence in favor of the electron transfer possibilities. The physical–chemical properties of polyimines p1 and p2 in the gaseous phase were compared using the electron density and electrostatic potential^60–62^. With the use of TD-DFT and TD-DFT/Gaussian ideas, electron systems of the gaseous phases of polyimine p1 and p2 may be computed based on the electron density (Fig. 7a,c). The potential diagrams in Fig. 7b,d indicate how the polyimine p1 and p2 gas-considerable phases could grow.

In quantum chemical calculations, the geometry of the ground state for multiple conformers was studied by selecting the lowest energy conformers depending on the harmonic vibrational frequency. Binding energies of the dimer’s were adjusted using counterpoise correction technique BSSE for the basis set superposition error. − 8516 and − 7659 kcal/mol are the binding energies for polyimine p1 and p2, respectively^63^.

**Figure 8:**
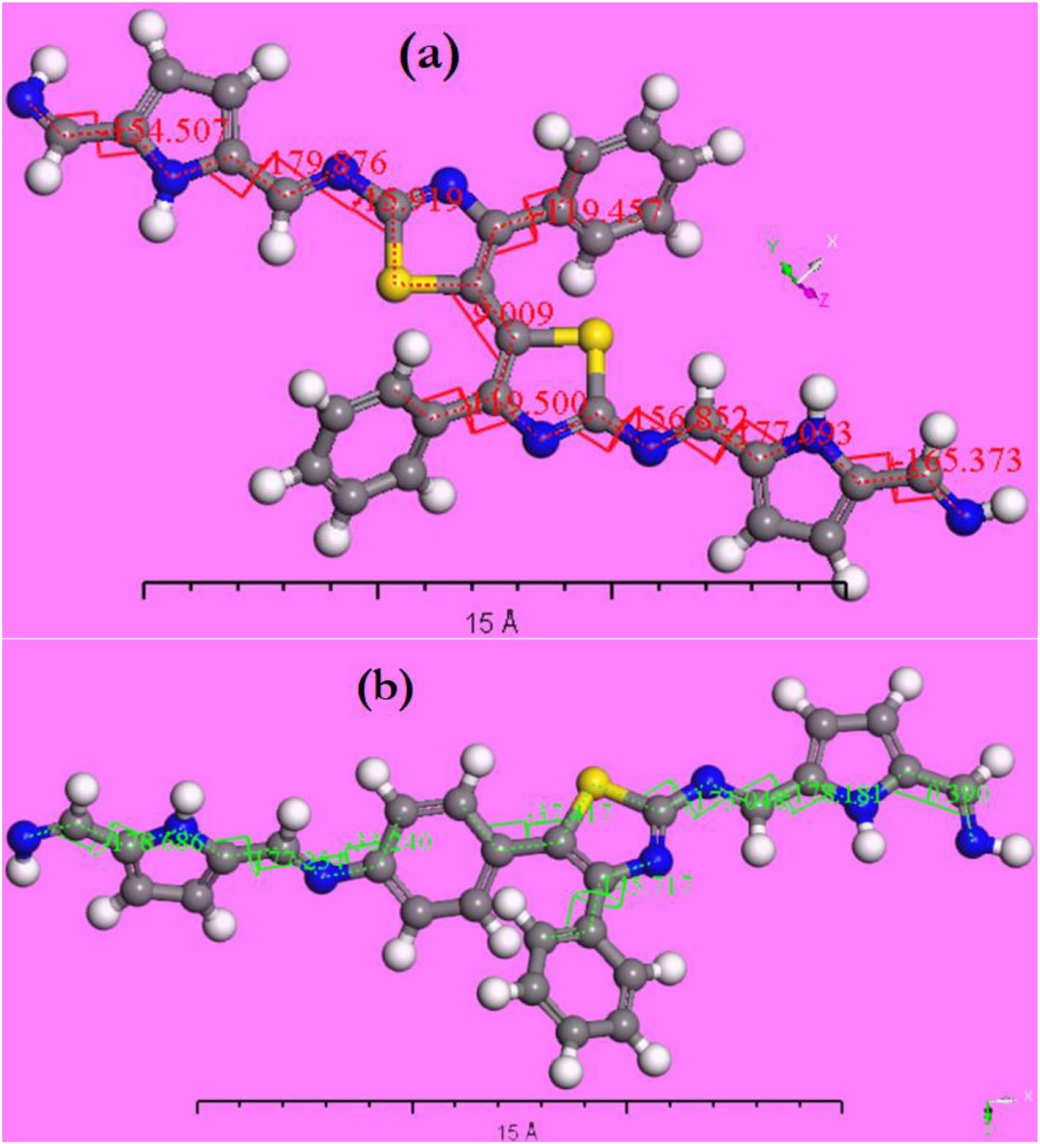
Conformers structure for polyimine (**a**) p1 and (**b**) p2.

Figure 8a,b represent the stable conformer structure for polyimine p1 and p2. In the case of polyimine p1 which includes N–C–C–N, N–C–N–C, C–C–C–N, S–C–C–C, C–C–C–C and S–C–N–C bonds the torsion corresponds to each bond was calculated. For the (N–C–C–N) the torsion angles found to be − 165.3°, − 177°, − 179.8° and − 154.5°. while for (N–C–N–C), (C–C–C–N), (S–C–C–C), (C–C–C–C) and (S–C–N–C) the torsion angles were − 156.8°, 119.5°, 9.009°, − 119.4° and − 15.9°, respectively. On the other hand, the torsion angles of polyimine p2 found to be − 178.6°, 177.2°, − 178.1° and 0.39° for (N–C–C–N) while the torsion angles of (C–C–N–C), (S–C–C–C), (C–C–C–N) and (S–C–N–C) found to be − 33.2°, − 37.4°, 145.7° and − 173°, respectively. The differences in the values of the torsion angles between p1 and p2 result in different values of radius of gyration. The radius of gyration was calculated for p1 and p2 and found to be 6.48 and 6.76 Ǻ, respectively.

**Figure 9:**
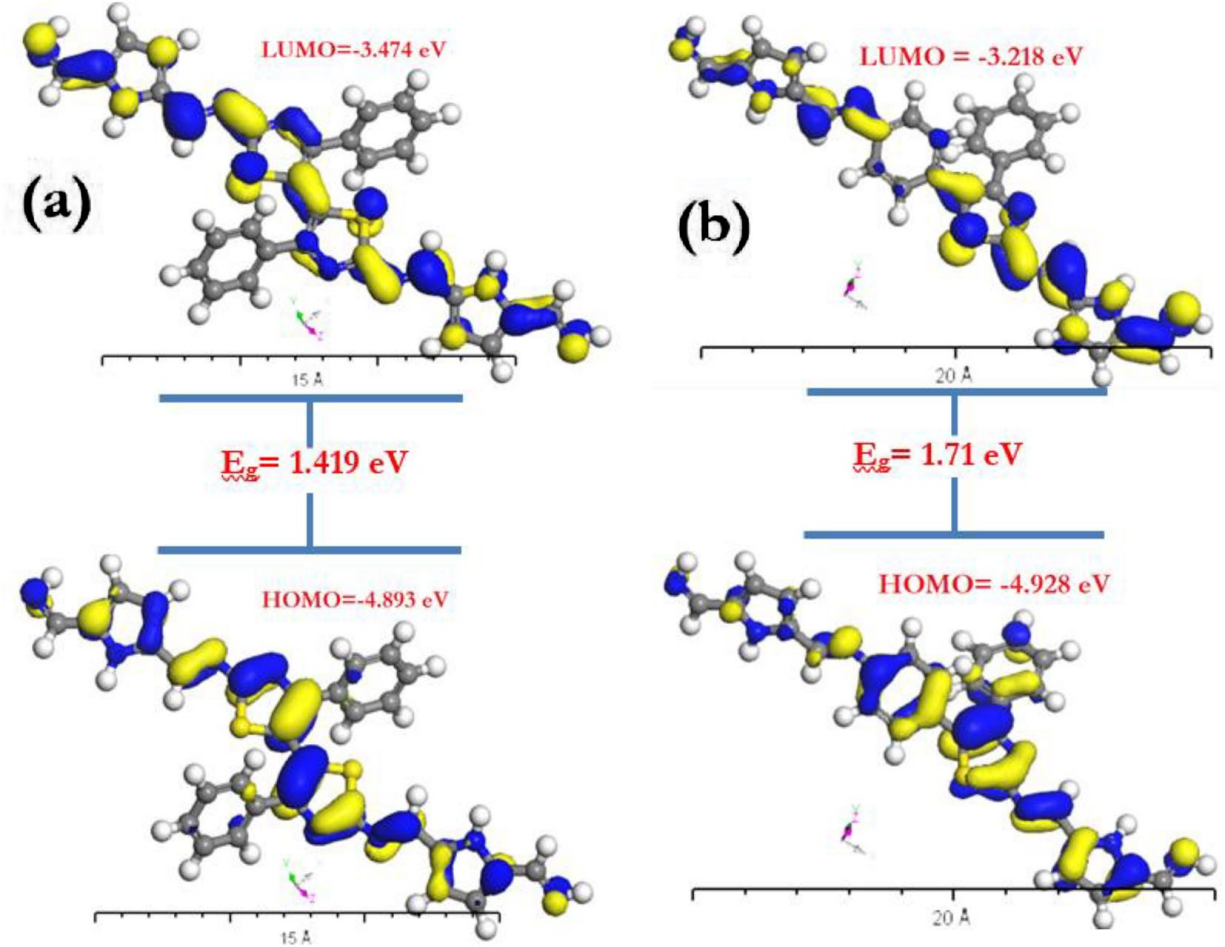
absolute values of HOMO and LUMO states energy for polyimine (**a**) p1 and (**b**) p2.

The energy gap was computed based on the difference between the highest occupied molecular orbital (HOMO) and the lowest unoccupied molecular orbital (LUMO), as illustrated in Fig. 9, and utilizing DFT-Dmol3 calculations. The simulations of the molecules' HOMO and LUMO states are directly relevant to the complex analysis of fragment molecular orbitals (FMOs). Using the equations of $(\mu = ( {E_{HOMO} + E_{LUMO} } )/2),$ $({\upeta } = (E_{LUMO} - E_{HOMO} )/2)$, $( {{\upchi } = - {\upmu }} )$, $( {S = 1/2{\upeta }} )$, $(\omega = \mu^{2} /2{\upeta })$, $({\upsigma } = 1/{\upeta })$ and $(\Delta N_{max} = - \mu /\eta )$ it is simple to calculate important physio-chemical parameters such as chemical potential (μ), softness (σ), global softness (*S*), global hardness (η), electronegativity (χ), global electrophilicity index (ω), and the maximum amount of electronic charge (Δ*N*_*max*_). Table 2 lists the values of E_HOMO_ and E_LUMO_ as well as the computed values of (*μ*), $({\upsigma })$, (*S*), $({\upeta })$, (χ), (ω), and $(\Delta N_{max} )$. While the key quantum chemical characteristic (ω) represents the energy stability of the molecule upon receiving extra electronic charge, E_HOMO_ and E_LUMO_ negative values reflect the stability of polyimine p1 and p2, respectively.

**Table 2:** Geometry constants for polyimine derivatives P1 and P2 as isolated molecules.

Compounds	E_HOMO_	E_LUMO_	E_g_	χ (eV)	µ(eV)	η (eV)	S (eV)	ω (eV)	ΔN_max_	σ
P1	− 4.893	− 3.474	1.419	4.183	− 4.183	0.709	0.705	12.33	5.899	1.410
P2	− 4.928	− 3.218	1.710	4.073	− 4.073	0.855	0.584	9.701	4.076	1.169

### Photophysical properties

**Figure 10:**
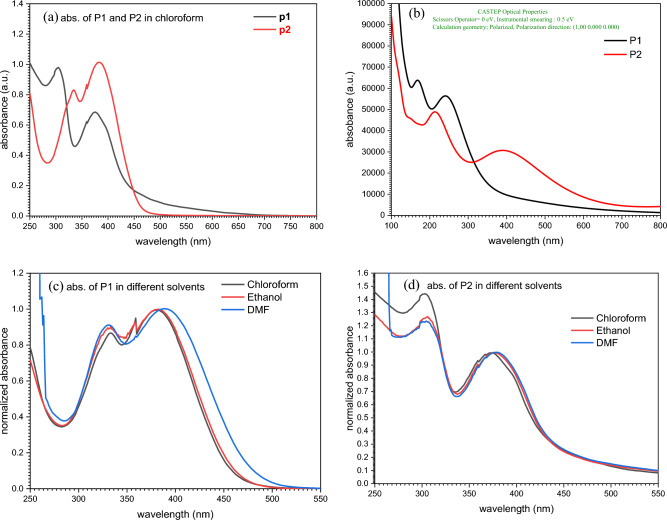
(**a**) The absorption spectrum for polyimine **P1** and **P2** dissolved in chloroform. (**b**) CASTEP absorbance simulation. (**c**,**d**) Absorbance of P1 and P2 in different solvents.

The polyimine P1 and P2's UV–Vis absorption spectra in chloroform solution showed absorption maxima at 372 nm and 381 nm, respectively, attributed to n–π* transitions of imine groups conjugated with aromatic nuclei^64^. Figure 10a shows the UV–Vis absorption spectra of the two polymers in chloroform solution. They exhibit maximum absorption maxima in the 250–330 nm range, which is typical of the π–π* transition in aromatic rings, while absorptions at longer wavelengths (340–430 nm) are attributed to the n–π* transitions of imine groups attached to aromatic nuclei^65^. Strong agreement between experimental and simulated data is shown in CASTEP optical characteristics (Fig. 10b), with only minor deviations.

Solvation effect of the absorbance of the polyimines P1 and P2 was studied using different solvents (chloroform, ethanol and DMF) as shown in Fig. 10c,d. It can figure out that the position of the absorption peak of n–π* transitions has been red shifted from 381 nm for chloroform to 383 and 389 nm for ethanol and DMF respectively. While the absorption peak of π–π* transition has been blue shifted from 333 nm for chloroform to 331 and 329 nm for ethanol and DMF respectively. On the other hand, polyimine P2 faces the same effect since the absorption peak of n–π* transition has been red shifted from 372 for chloroform to 377 and 379 nm for ethanol and DMF respectively. While the absorption peak of π–π* transition has been blue shifted from 302 nm for chloroform to 306 and 304 nm for ethanol and DMF respectively. The position of the absorption bands of P1 and P2 in different solvents are tabulated in Table 3.

**Table 3:** optical data of polyimines.

Sample	Solvent	λ_abs_ (nm)	λ_emi_	PLQY%
P1	Chloroform	333, 381	505	34.2
	Ethanol	331, 383	523	32.6
	DMF	329, 389	540	58.2
	Film	385	540, 688	59
P2	Chloroform	302, 372	491	24.1
	Ethanol	306, 377	500	17
	DMF	304, 379	498	20
	Film	389	525, 706	42.7

The energy gap (also known as the energy gab) between the highest occupied molecular orbit (HOMO) and the lowest unoccupied molecular orbit (LUMO) was estimated optically using the Tauc relation as described in^66,67^:

$$\left( {\alpha h\upsilon } \right)^{2} = B\left( {h\upsilon - E_{g} } \right)$$

**Figure 11:**
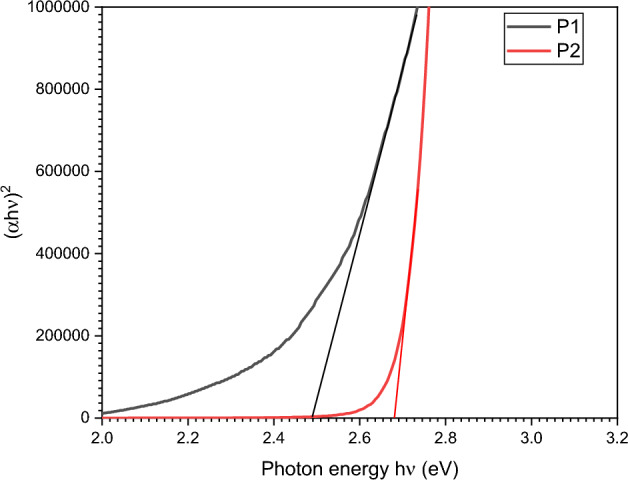
Experimental calculations of bandgap energies for polyimines P1 and P2.

The absorption coefficient was computed as $\alpha = 2.303A/x$, where x is the sample thickness and $h\upsilon$ is the photon energy. Figure 11 illustrates the Tauc relation graphically for polyimines p1 and p2, from which the direct optical energy gap was determined, as well as the intercept between the extrapolated line and axis. For P1 and P2, the direct optical energy gap was discovered to be 2.49 and 2.68 eV, respectively. The optical energy gap values discovered to be consistent with the substances used in blue and white light emitting diodes in the literature^68,69^.

**Figure 12:**
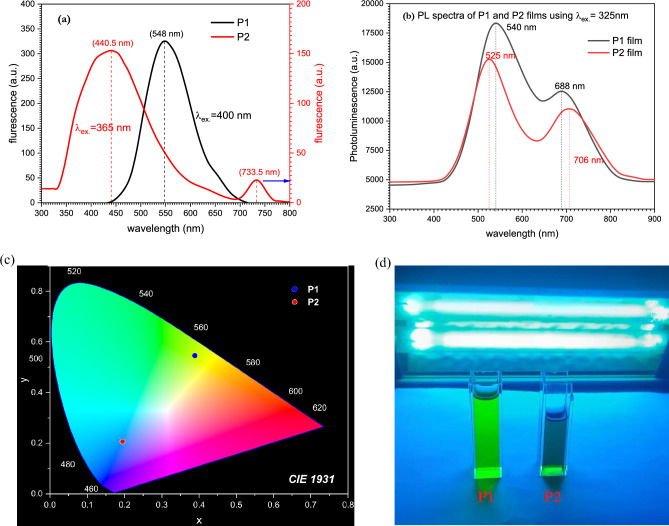
emission spectra for polyimine p1 and p2.

Photoluminescence properties of polyimine derivatives were studied in terms of fluorescence and laser photoluminescence for liquid and solid samples respectively. P1 and P2 were dissolved in DMF with a concentration of 1 × 10^−4^ mol/L and the fluorescence spectra were recorded using Shimadzu RF-1501 spectrofluorometer as shown in Fig. 12a. On the other hand, the spin coating method was used to deposit the polymers on a glass substrate to record the laser photoluminescence for the solid state of the polyimine derivatives (Fig. 12b). A He–Cd laser with 325 nm wavelength and 150 mW power was used to measure the emission spectra, which was recorded using HoRiBA (IHR 320) spectrum analyzer with a computerized CCD camera. Figure 12a shows the fluorescence spectra of polyimine P1 and P2 using excitation wavelength of 400 and 365 nm for P1 and P2 respectively. It can be investigated that the emission profile is much different for the two derivatives. For polyimine P1 there is only one emission peak located at 538 nm, while for polyimine P2 there is a main emission peak located at 440.5 nm and there is another weak peak that appeared at 733.5 nm. Also, it can be figured out that the emission intensity for polyimine P1 is much higher than that of polyimine P2. On the other hand, the main emission peak for P2 is much broader than that of P1. Photoluminescence spectra of polyimine P1 and P2 films are shown in Fig. 12b. The emission spectra of P1 and P2 are nearly identical, as there is a major emission at 540 and 525 nm for P1 and P2, respectively. Another emission peak, however, may be found at 688 and 706 nm for P1 and P2, respectively. The emission color of polyimine P1 and P2 dissolved in DMF were analyzed using Commission Internationale de l'Eclairage (CIE) graphs to illustrate the emission colour of the two polyimine derivatives (Fig. 12c) it can figure that for polyimine P1 the digital photograph is located at the coordinates of (0.489, 0.547), while for polyimine P2 the digital photograph is located at (0.194, 0.205). a significant difference in the emission colour is figured out since the emission colour for P1 is located in the yellow green colour while for P2 it is located in the blue colour. The significant emission profile and changes between polyimine P1 and P2 give them potential to be used in photoluminescence applications such as light emitting diodes and laser dye. Figure 12d shows the photograph of the emission of the polyimines P1 and P2 under the irradiation of UV lamp to show the emission colour of P1 and P2 which found to be compatible with CIE graph where the P1 found to emit yellow green electromagnetic waves while P2 emits blue electromagnetic waves.

**Figure 13:**
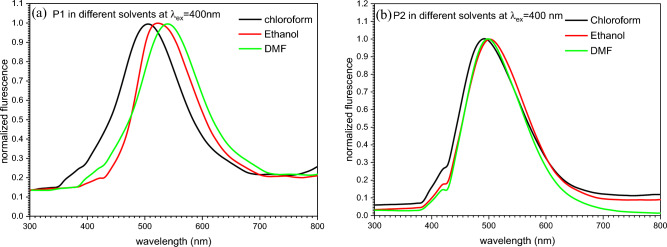
Emission spectra of (**a**) P1 and (**b**) P2 in different solvents.

Emission spectra of P1 and P2 was recorded in different solvents to study the effect of solvation on the emission of the polymers as shown in Fig. 13a,b. The fluorescence measurements were applied using excitation wavelength of λ_ex_ = 400 nm. It is obvious that, the position of λ_max_ found to be red shifted by changing the solvent from chloroform to ethanol and DMF for the two polymers. For P1 the position of λ_max_ found to be shifted from 505 nm for chloroform to 523 and 540 nm for ethanol and DMF respectively. On the other hand, λ_max_ was shifted from 491 nm for chloroform to 500 and 498 nm for ethanol and DMF respectively in case of P2.

The measured absorbance and emission data was then used to measure the photoluminescence quantum yield of the used polymers relative to fluorescein standard dye using the equation^70,71^:

$$\emptyset_{x} = \emptyset_{st} \left( {\frac{{A_{st} }}{{A_{x} }}} \right)\left( {\frac{{F_{x} }}{{F_{st} }}} \right)\left( {\frac{{n_{x}^{2} }}{{n_{st}^{2} }}} \right)\left( {\frac{{D_{x} }}{{D_{st} }}} \right)$$

where Ø, A, F, n and D are the quantum yield, absorbance, fluorescence, refractive index and dilution respectively and x and st denotes to the sample and the standard respectively. The calculated values of the quantum yield of polyimines P1 and P2 are listed in Table 3.

Photophysical properties of polyimine derivatives P1 and P2 summarized in Table 3 shows medium emission in diluted solution where the PLQYs ranges between 34.2 and 58.2% for P1 and between 17 and 24.1% for P2 with no relevant solvatochromism in dependence of solvent polarity. While the stokes shift was found to be about 120–150 nm for both polymers in the form of solution and film. On the other hand, both polymers were found to have strong emission while they are in the solid form which indicated the aggregation induced emission (AIE) nature of the polymers which would make the applicable in optoelectronic applications^72^.

## Conclusions

Two polyimine derivatives were obtained by the polymerization of thiazole and pyrrole-based Schiff bases using a conventional heating besides the environmentally friendly benign techniques. Static light scattering technique was deployed to determine the molecular weight of the synthetic polymers. It is obvious that the microwave irradiation approach produces good results with the molecular weight of polyimines P1 and P2. FT-IR and ^1^H NMR spectra were measured to confirm the synthetic process, while the thermal stability of the synthetic polymers was investigated by TGA method. TD-DFT simulations were performed to geometrically optimize the chemical structure of the synthetic polymers instead of studying their conformer stable structure. Optical properties of the synthetic polymers were studied by UV–Vis spectrophotometer absorbance spectra and showed a main absorption bands at 375 and 382 nm for polyimine P1 and P2 respectively. While the energy gap was found to be 2.49 and 2.68 eV for polyimine P1 and P2 respectively. The photoluminescence spectra were evaluated for the two polymers and found to have better photoluminescence as a solid material than in diluted solution. On the solid samples, there have been large Stokes Shifts (over 100 nm) and PLQYs above 50%. The polyimines are now very promising as simple and affordable solid-state emitters as a result of these findings.

## Data Availability

All data generated or analyzed during this study are included in this published article.
